# Sporadic unilateral papillary renal cell carcinoma masquerading pyelonephritis: A case report

**DOI:** 10.1002/ccr3.6976

**Published:** 2023-02-19

**Authors:** Mohan Nayak Guguloth, Juhi Manzoor, Sai Kiran Kuchana, Tarun Kumar Suvvari, Anthony Reddy Gopu, Vikram Das Kunden, Sashank Bhattarai, Ayush Anand

**Affiliations:** ^1^ Department of General Surgery Kakatiya Medical College Warangal India; ^2^ Kakatiya Medical College Warangal India; ^3^ Rangaraya Medical College Kakinada India; ^4^ BP Koirala Institute of Health Sciences Dharan Nepal

**Keywords:** case report, nephrectomy, papillary, pyelonephritis, renal cell carcinoma

## Abstract

Hidden renal cell carcinoma (RCC) is not evident during severe inflammation such as acute pyelonephritis. We presented a 62‐year‐old male presenting with features of obstructive pyelonephritis managed with simple nephrectomy. However, the histopathology findings suggested unilateral papillary RCC. Our case showed that RCC might present with features of obstructive pyelonephritis.

## Abstract 2

Severe inflammation, such as obstructive pyelonephritis, may masquerade RCC, which may delay the cancer diagnosis and lead to improper management.

## 1 INTRODUCTION

The global incidence of renal cell carcinoma (RCC) is 4.4 per 100,000, with an increased cumulative risk of incidence in males and the peak age of presentation between 60 and 70 years.^1^ Papillary RCC, a subtype of RCC, accounts for about one‐eighth of all cases and is frequently associated with advanced kidney disease.^2^ Rarely, RCC presents with features suggestive of acute pyelonephritis.^3^ RCC related to inflammatory conditions such as abscess formations or pyelonephritis may delay the cancer diagnosis and lead to improper management. Furthermore, inflammatory and neoplastic systemic signs overlap, and radiologic studies may sometimes fail to differentiate between these two disease entities.^3^ Herein, we present the case of a 62‐year‐old male with left obstructive pyelonephritis, later identified as unilateral sporadic renal papillary cell carcinoma.

## 2 CASE REPORT

A 62‐year‐old Asian male presented with left lumbar pain and burning micturition for 2 months. The pain was of moderate severity, dragging type and radiating to the loin. Also, he had increased frequency and urgency of micturition of the same duration. The patient did not have a fever. His medical, family and personal history were unremarkable. The patient was hemodynamically stable with a temperature of 98.4°F. Physical examination revealed left costovertebral angle tenderness with no palpable mass over the abdominal region. The rest of the systemic examinations were normal.

**FIGURE 1:**
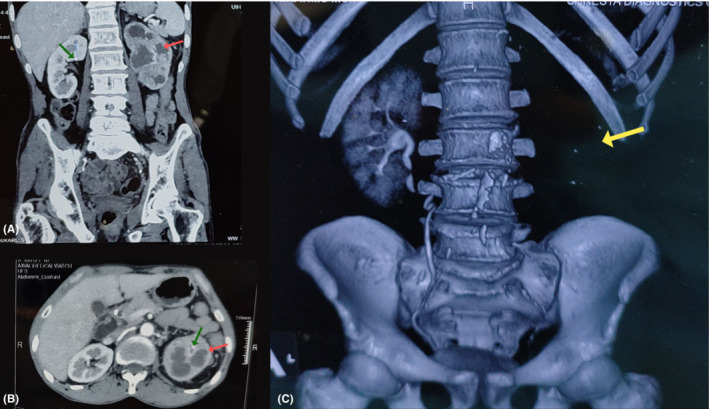
(A). Axial section of CECT scan revealing enlarged left kidney 109 × 69mm with mild dilatation of pelvicalyceal system and perinephric fat inflammation (red arrow showing hypodense lesion, green arrow showing hyperdense lesion). (B). Coronal section of CECT scan revealing enlarged left kidney 109 × 69mm with mild dilatation of pelvicalyceal system and perinephric fat inflammation. (C). Post‐contrast study showing decreased irregular enhancement pattern and no excretion in the left kidney (yellow arrow showing non‐excreting left kidney).

**FIGURE 2:**
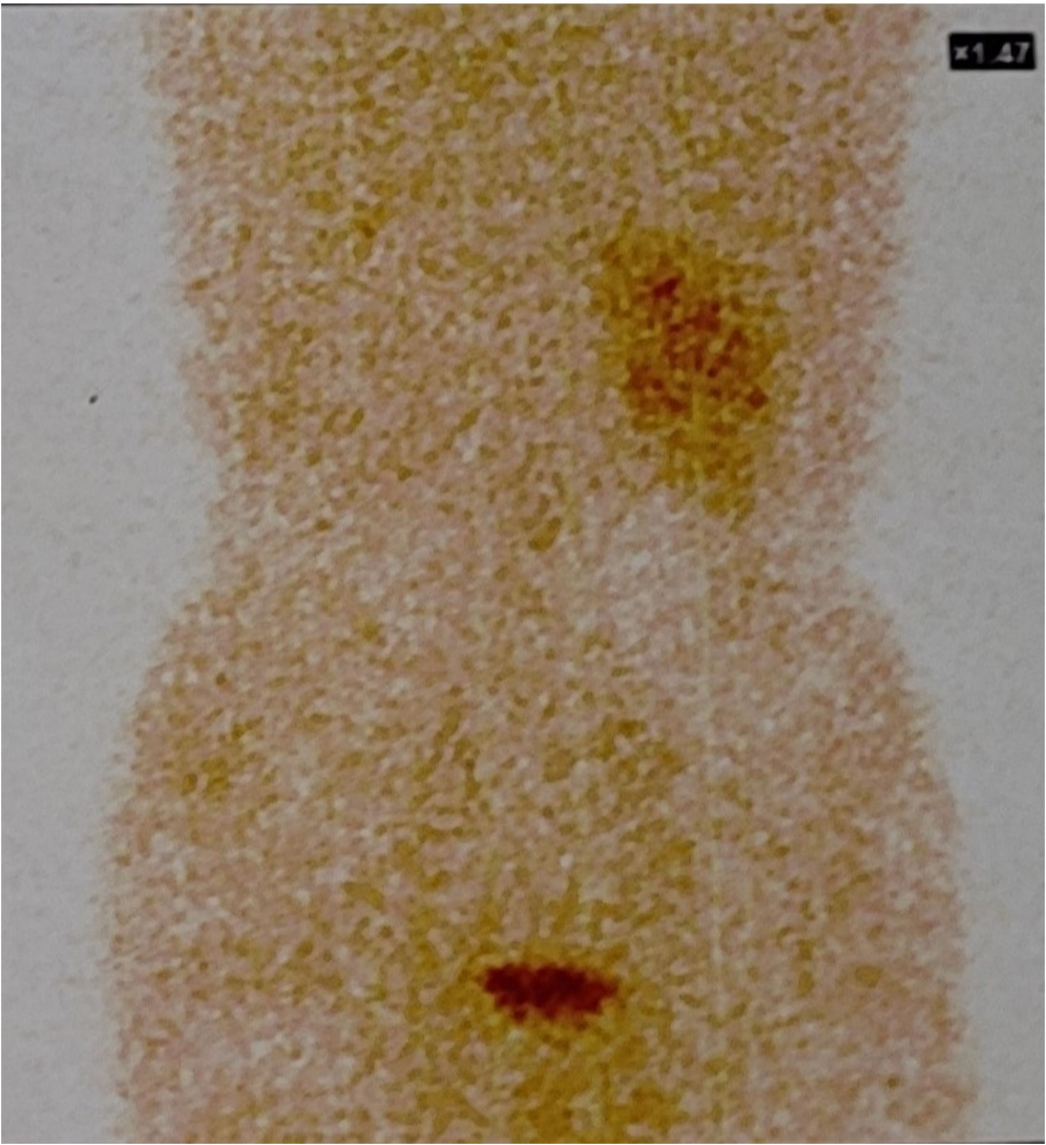
DPTA Renogram showing no significant tracer uptake in the left kidney.

Laboratory investigations showed hemoglobin of 9.8 g/dL, total leukocyte count of 14,800 cells/mm^3^, platelet count of 4 lacs/mm^3^, serum urea of 25 mg/dL, and serum creatinine of 1 mg/dL. And, urinalysis revealed +1 for glucose and protein, with pus cells 10‐20/HPF. Contrast‐enhanced computed tomography (CECT) of the abdomen and pelvis (Figure 1) showed the left kidney measuring about 109 × 69 mm with mild pelvicalyceal system dilatation, hypodense lesions (maximum size of 23 × 28 × 10 mm), perinephric fat inflammation, and multiple calculi in both right and left kidneys (maximum size of 11 × 8 × 6 mm). A post‐contrast study showed irregular enhancement patterns; no excretion was noted in the left kidney (Figure 1). We diagnosed left pyelonephritis in our patient. We further evaluated the patient with a radioisotope renogram using 99mTc‐DTPA (Diethylene triamine penta acetate) injection. The radioisotope renogram revealed a non‐functioning left kidney (Figure 2).

**FIGURE 3:**
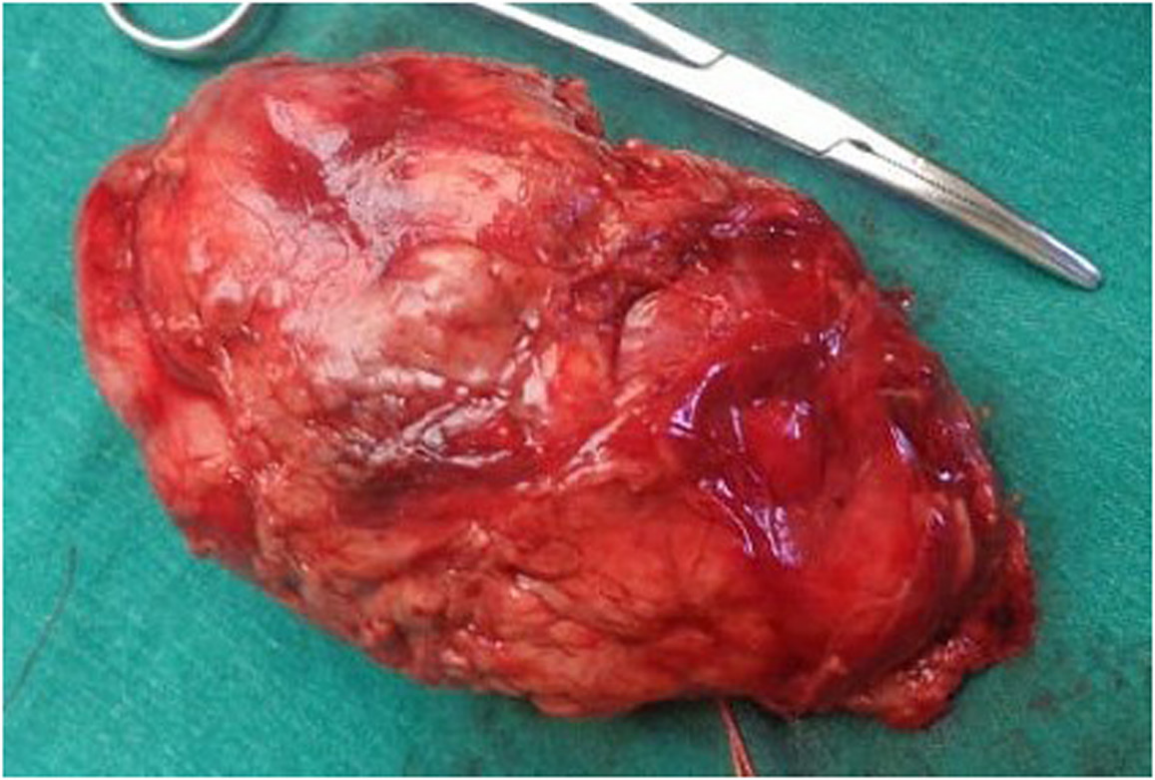
Gross morphology of resected specimen.

Following this, we did a simple nephrectomy under general anesthesia. While keeping the patient in the right lateral position, we gave a cutting incision on the left 11th rib. Then we dissected the external oblique muscle, transected the internal oblique muscle, and cut the transversus abdominis muscle. After that, we opened the renal fascia and did the blunt dissection of perirenal fat and psoas muscle. Then the kidney was identified and separated from the surrounding Gerota's fascia (Figure 3). Also, the ureter was identified and mobilized to the hilum. Following this, we did en mass ligation of the kidney at the hilum, and the ureter was ligated and cut. Hemostasis was secured, and the wound was closed in layers. Following the surgical management, we did a histopathology examination of the resected specimen. The histopathology revealed papillae with fibrovascular stalks lined by tumor cells and an accumulation of foam cells in the papillary stalk. There were large tumor cells, cuboidal to low columnar, with abundant acidophilic cytoplasm. And, the cytoplasm clearing was apical with large spherical nuclei and prominent nucleoli. Also, a fibrovascular capsule lined the lesion. These findings suggested a Grade 3 papillary RCC. TNM staging of renal cell carcinoma was T1 N0 M0. Following the surgical management, the patient was discharged after 48 hours. However, we could not obtain long‐term follow‐up information as the patient was lost to follow‐up.

## 3 DISCUSSION

Papillary RCC is a malignant renal parenchymal tumor showing a 5:1 male predilection with the usual incidence in the sixth to seventh decades.^4^, ^5^ Similar to this, our patient was a male presenting in his early 60s. Pyelonephritis is usually a clinical diagnosis classically presenting with flank pain and fever.^6^, ^7^ Clinical presentation and urinalysis are sufficient to reach a diagnosis. Our patient had flank pain with pyuria on urinalysis. So, we made a provisional diagnosis of pyelonephritis. For further evaluation of the severity and complications of pyelonephritis, CECT of the abdomen and pelvis is useful.^8^ In our case, we worked up initially using CECT. We diagnosed pyelonephritis according to the patient's presentation and radiological findings on the CECT scan. However, due to the non‐specificity of these lesions, achieving a differential diagnosis on radiological examination can be difficult before surgery.^8,9^ A retrospective study further highlighted that 0.2%–6.5% of papillary renal cell carcinomas cases masquerade renal cysts and other inflammatory conditions on radiological evaluation.^10^ This was quite evident in our case, as the CECT findings suggested pyelonephritis. However, the histopathology suggested Grade 3 papillary renal cell carcinoma. Similar cases have been reported where unilateral or bilateral RCC has presented as pyelonephritis in elderly males. Karthikeyan et al. reported sporadic bilateral papillary RCC masquerading as bilateral multifocal pyelonephritis.^11^ Also, Boukhannous et al. reported a unilateral RCC masquerading as pyelonephritis.^3^ Like Boukhannous et al., our patient had unilateral papillary RCC presenting with features of pyelonephritis. Our management was focused on obstructive pyelonephritis. Due to a non‐functioning unilateral kidney, we aimed for a simple nephrectomy.^12^, ^13^ As the patient was lost to follow‐up, we could not obtain long‐term follow‐up information.

## 4 CONCLUSION

Our case illustrates that a surgeon should be vigilant while treating a case of obstructive pyelonephritis and always consider a nuclear scan or renal biopsy to look for a possible masquerading renal cell carcinoma.

## AUTHOR CONTRIBUTIONS

**Mohan Nayak Guguloth:** Writing – original draft; writing – review and editing. **Juhi Manzoor:** Writing – original draft; writing – review and editing. **Sai Kiran Kuchana:** Writing – original draft; writing – review and editing. **Tarun Kumar Suvvari:** Writing – original draft; writing – review and editing. **Anthony Reddy Gopu:** Writing – original draft; writing – review and editing. **Vikram Das Kunden:** Writing – original draft; writing – review and editing. **Sashank Bhattarai:** Writing – original draft; writing – review and editing. **Ayush Anand:** Conceptualization; project administration; supervision; visualization; writing – original draft; writing – review and editing.

## FUNDING INFORMATION

The authors did not receive any funding for the manuscript.

## CONFLICT OF INTEREST STATEMENT

The authors have no conflict of interest to declare.

## GUARANTOR

SKK is the guarantor of the manuscript.

## INFORMED CONSENT

A written informed consent was obtained from the patient based on the journal's policies.

## Data Availability

All the Data pertaining to this case report is available within this manuscript.
